# Thermography in clinical ophthalmic oncology

**DOI:** 10.5935/0004-2749.20210004

**Published:** 2025-02-02

**Authors:** Anna Modrzejewska, Łukasz Cieszyński, Daniel Zaborski, Mirosław Parafiniuk

**Affiliations:** 1 I Department of Ophthalmology, Pomeranian Medical University, Szczecin, Poland; 2 Faculty of Computer Science and Information Technology, West Pomeranian University of Technology, Szczecin, Poland; 3 Faculty of Biotechnology and Animal Husbandry, West Pomeranian University of Technology, Szczecin, Poland; 4 Forensic Medicine Institution, Pomeranian Medical University, Szczecin, Poland

**Keywords:** Thermography, Uveal neoplasm, Melanoma, Neoplasm metastasis, Eye neoplasm/secondary, Hemangioblastoma, Termografia, Neoplasias uveais, Melanoma, Metás tases neoplásicas, Neoplasias oculares/secundário, Hemangioblastoma

## Abstract

**Purpose:**

The aim of this study was to present our own experience with the use of thermography as a complementary method for the initial diagnosis and differentiation of intraocular tumors, as well as for the evaluation of the efficacy of treatment of intraocular melanomas.

**Methods:**

The study group comprised 37 patients with intraocular tumors, including 9 with uveal melanoma, 8 with uveal melanoma after I^125^ brachytherapy, 12 with a focal metastasis to the uvea, and 8 with retinal capillary hemangioblastoma. A FLIR T640 camera was used to capture images in the central point of the cornea, eye area, and orbital cavity area.

**Results:**

Eyes with uveal melanoma had higher temperature compared with the fellow normal eye of the patient in the range of all measured parameters in the regions of interest. In the group of patients with melanoma after unsuccessful brachytherapy, higher temperature was observed at the central point of the cornea. In patients with tumor regression, all measured parameters were lower in the affected eye. We observed lower temperatures in the range of all tested parameters and areas in eyes with choroidal metastases. Eyes with diagnosed intraocular hemangioblastoma were characterized by higher parameters for the regions of interest versus eyes without this pathology.

**Conclusions:**

A thermographic examination of the eye can be used as an additional first-line diagnostic tool for the differentiation of intraocular tumors. Thermography can be a helpful tool in monitoring the treatment outcome in patients with intraocular melanoma.

## Abstract (pt)

**Objetivo:**

O objetivo deste estudo foi de apresentar a nossa experiência no uso da termografia como método complementar para o diagnóstico inicial e a diferenciação de tumores intraoculares, bem como para a avaliação da eficácia do tratamento de melanomas intraoculares.

**Métodos:**

O grupo estudado compunha-se de 37 pacientes com tumores intraoculares, sendo 9 com melanoma uveal, 8 com melanoma uveal após braquiterapia com I^125^, 12 com metástases focais na úvea e 8 com hemangioblastoma capilar retiniano. As imagens do ponto central da córnea, da área do olho e da área da cavidade orbital foram obtidas com uma câmera FLIR T640.

**Re sultados:**

Os olhos dos pacientes com melanoma uveal tinham temperaturas mais elevadas do que as dos olhos normais dos mesmos, em toda a faixa dos parâmetros medidos nas regiões de interesse. No grupo de pacientes com melanoma após braquiterapia mal sucedida, encontrámos temperaturas maiores no ponto central da córnea. Nos pacientes com regressão do tumor, todos os parâmetros medidos foram menores no olho acometido. Encontrámos temperaturas mais baixas em toda a faixa dos parâmetros testados e das áreas medidas nos olhos com metástases na coroide. Os olhos com hemangioblastoma intraocular diagnosticado caracterizaram-se por parâmetros mais elevados nas regiões de interesse, em comparação com olhos sem essa patologia.

**Conclusões:**

O exame termográfico do olho pode usar-se como ferramenta de diagnóstico adicional de triagem na diferenciação de tumores intraoculares. A termografia pode ser uma ferramenta útil no acompanhamento do desfe cho do tratamento em pacientes com melanoma intraocular.

## INTRODUCTION

Thermography is an imaging technique which detects radiation in the long-infrared range of the electromagnetic spectrum emitted by various objects, including human tissues. Radiation is emitted at temperatures above absolute zero, i.e., -273.15°C or 0 K(1). A thermographic camera shows the exact value and distribution of the temperature of the examined surface, which depends on the vascularization and metabolism of the tissue. This technique captures thermal images, which are actually visual displays of the amount of infrared energy transmitted, emitted, or reflected by the tissue.

The search for thermal imaging applications in medicine was initiated in the 20th century. Thus far, most attempts focused on the use of this technique in the detection of breast cancer. Studies have demonstrated that thermography can be used to define the boundary between the area of normal tissue and the area of tissue affected by cancer^(2,3)^. Thermography is a safe, noninvasive, and reproducible imaging technique that can be used for screening in the early diagnosis of cancer. However, this diagnosis has to be confirmed by mammography, ultrasonography (US), and histopathological examination.

It is often difficult to differentiate between malignant and benign intraocular tumors or to determine the tumor grade and its type, especially in cases of amelanotic changes and those accompanied by retinal detachment. The diagnosis usually relies on the examination of morphological changes in the fundus, US in A and B projections, color Doppler imaging (CDI), optical coherence tomography, fluorescein angiography (FA), or indocyanine green angiography^(4)^. The diagnosis and qualification for treatment strongly depend on the experience of the examiner or person assessing the results of the tests. Aside from histopathological examination, there are very few objective methods for differentiating neoplastic lesions and evaluating the efficacy of treatment in patients with intraocular tumors.

This article presents our own experience with the use of thermography as a complementary method for the initial diagnosis and differentiation of intraocular tumors, as well as for the evaluation of the efficacy of treatment of intraocular melanomas.

## METHODS

This was an analytical, prospective, cross-sectional study. The study group comprised patients with suspected intraocular tumors treated at the Ophthalmology Department, Pomeranian Medical University (Szczecin, Poland), from 2016 to 2018 (convenience sampling). A total of 37 patients were diagnosed with intraocular tumors based on clinical examination and imaging studies (i.e., US, CDI, FA, and optical coherence tomography). The study group included 9 patients with uveal melanoma, 8 patients with uveal melanoma after 6-month brachytherapy with I^125^ (4 without improvement after treatment and 4 in remission), 12 patients with a focal metastasis to the uvea (4 with primary breast cancer, 1 with lung cancer, and 7 with primary cancer of unknown location), and 8 patients with retinal capillary hemangioblastoma with Von Hippel-Lindau syndrome. Remission of tumor after brachytherapy involves a decrease in tumor height and increase in internal reflectivity^(5)^. Patients with ophthalmic conditions (e.g., dry eye syndrome, glaucoma, ocular inflammation, age-related macular degeneration) and fever, potentially disturbing the measurement of thermal emission on the eye surface were excluded from the study. An ophthalmic interview was conducted in the study group. A FLIR T640 thermographic camera was used to capture facial images (thermographic and optical) of each patient in three replications, perpendicularly to the examined area, 3 s after blinking, at a distance of 1 m after resting for 15 min in the examination room. Room temperature and air humidity were relatively stable, and the examination room was isolated from external sources of heat, air conditioning, or solar radiation. Images were captured at short intervals (every 1 s), which reduced the impact of variability on the external environment. The average of three measurements in each patient did not exhibit a significant standard deviation. The test area (eyeball) did not change its metabolic activity over time; hence, it can be assumed that the temperature of the test area was constant throughout the examination.

The protocol was approved by the Bioethics Committee of the Medical University (Approval No. KB-0012/ 141/15).

**Figure 1:**
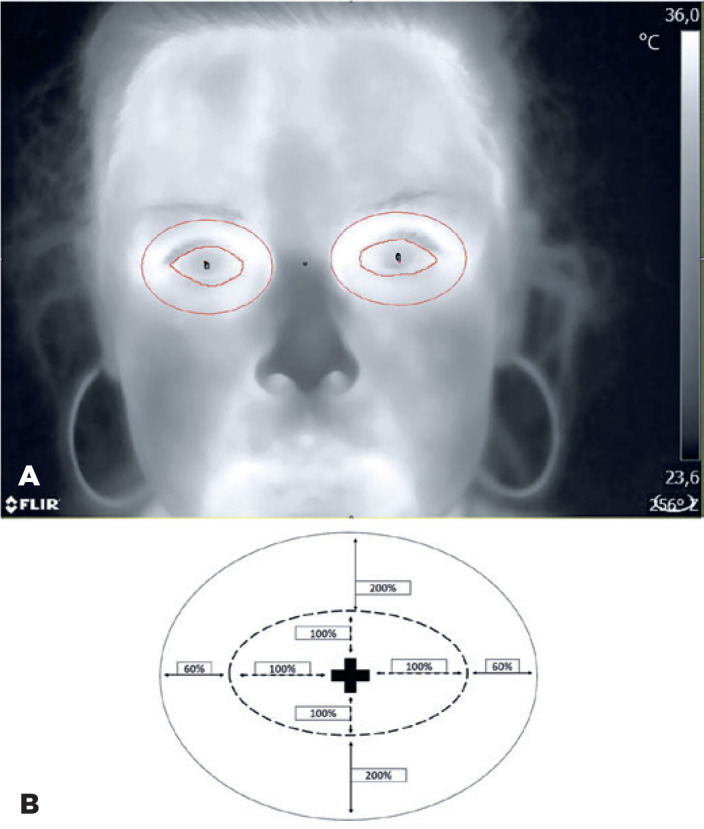
A) The analyzed area of the eye delineated as the surface between the eyelids and the orbital cavity delineated on the optical image and superimposed on the thermal image. B) Margins of the orbital cavity in the form of an ellipsoidal area with a minor axis equal to a double distance between the center of the pupil and the upper edge of the eye and the major axis equal to 0.6 of the distance between the center of the pupil and the left/right margin of the eye.

Images were processed using the ImageJ software for image analysis in the MATLAB environment. The following regions of interest were analyzed: the central point of the cornea in the left and right eyes, left and right eyes (area), and left and right orbital cavities (area). The area of the eye was delineated manually after the superimposition of the thermographic image as the surface of the eye between eyelids and the orbital cavity (Figure 1A). The last area was delineated as an ellipse with a minor axis equal to a double distance between the center of the pupil and the upper edge of the eye, and the major axis equal to 0.6 of the distance between the center of the pupil and the left/right margin of the eye (Figure 1B), and superimposed onto the optical image.

All thermal imaging tests and digital image analysis were conducted by a single investigator. The investigators were not aware which eye was affected or which type of tumor was diagnosed. Informed consent for the capture and publication of images was provided by all participants in this study.

For the statistical analysis of the results obtained from individual groups, Student’s t-test for paired samples was employed to compare the mean values of in dividual temperature parameters (mean, standard deviation, minimum, maximum, median, and mode) in the affected and normal eyes.

The effect of age, sex, tumor type, and the presence of an intraocular tumor (affected vs. normal eye) on the median temperature was analyzed using a general linear model with repeated measures.

All calculations were performed using the Statistica software (v. 13; Dell Inc., Tulsa, OK, USA). A p≤0.05 indicated statistical significance.

## RESULTS

The characteristics of the study group are presented in table 1. The results of the thermographic analysis of intraocular tumors in the study group (without classification to specific types of tumor) are presented in table 2. The median temperature was selected for further study due to its desired properties (i.e., insensitivity to outliers and strong statistically significant correlations with the remaining temperature parameters). There were no significant differences between the affected and normal eyes; however, the affected eyes had a higher temperature than the normal eyes.

**Table 1:** Demographic characteristics of examined patients

Group	Description	n	Age (years)		Female		Male	
			Mean	SD	n	%	n	%
1	Melanoma without treatment	9	73.89	9.78	2	22.22	7	77.78
2	Melanoma after treatment (relapse)	4	67.50	19.23	2	50.00	2	50.00
3	Melanoma after treatment	4	57.50	12.07	1	25.00	3	75.00
	(regression)							
4	Metastasis	12	67.92	12.77	9	75.00	3	25.00
5	Retinal capillary hemangioblastoma	8	44.50	22.05	5	62.50	3	37.50
Total		37	63.14	18.11	19	51.35	18	48.65

n= number of patients; SD= standard deviation.

**Table 2:** Mean, median, and standard deviations for temperatures measured at the three regions of interest of affected and normal eyes (*n*=37)

ROI	Variable	Affected		Normal	
		Mean	SD	Mean	SD
Central point of the cornea	Mean	33.63	1.07	33.54	1.14
Area of the eye	Median	34.31	0.90	34.27	0.93
Area of the orbital cavity	Median	34.44	0.81	34.37	0.82

Median denotes the middle value in a series arranged from the lowest to the highest, separating the same number of observations on both sides. ROI= region of interest; SD= standard deviation.

**Figure 2:**
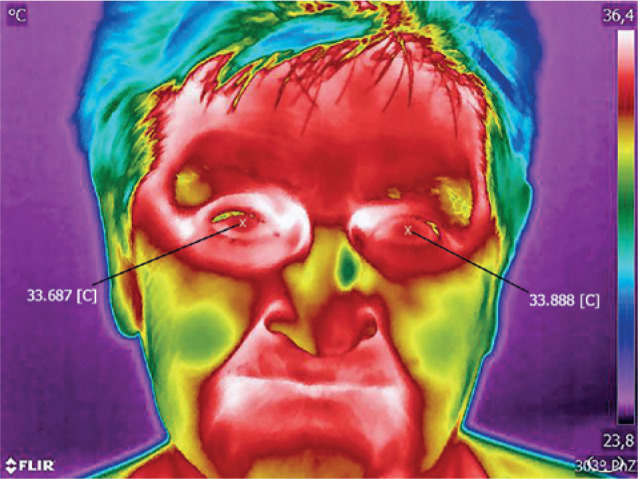
Uveal melanoma of the right eye.

**Figure 3:**
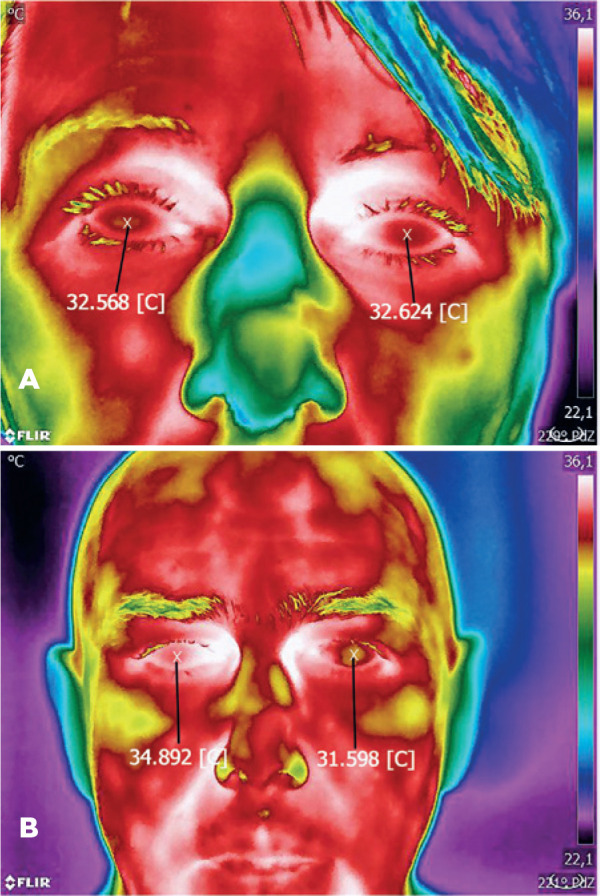
A) Uveal melanoma in the left eye after unsuccessful brachytherapy. B) Uveal melanoma in the left eye after successful brachytherapy.

**Figure 4:**
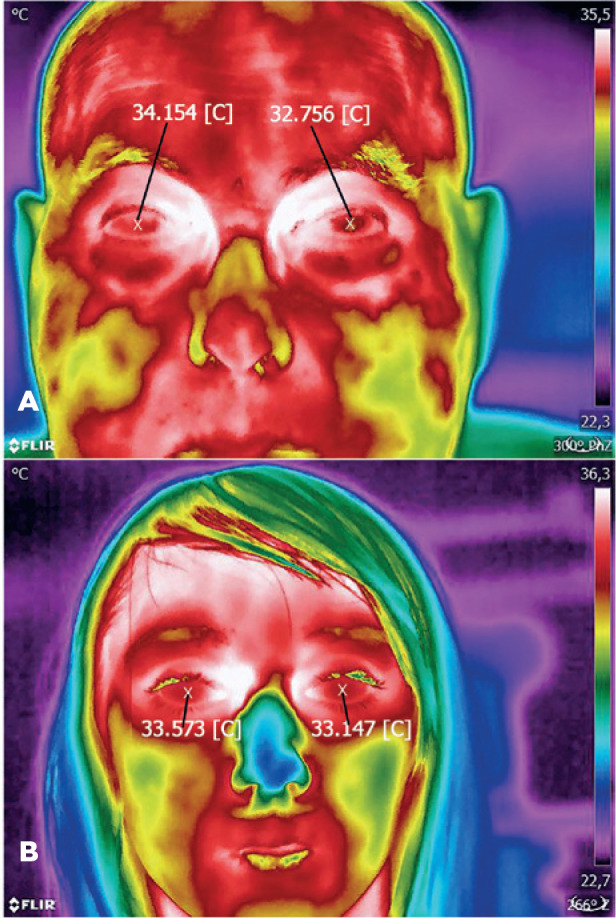
A) Focal metastasis to the uvea in the left eye. B) Retinal capillary hemangioblastoma in the right eye.

Selected thermal images of patients from specific subgroups are presented in figures 2-4. The results of the thermographic analysis of intraocular tumors with classification to specific types of tumor (five groups) are presented in table 3. Significant differences in the mean values (p≤0.05) between the affected and normal eyes were observed in Group 1 (uveal melanoma) for the temperature at the central point of the cornea, median and mode temperature of the eye, and mean, median, and mode of the temperature of the area of the orbital cavity; standard deviation for the temperature of the area of the eye in Group 3 (melanoma after successful treatment); and mean and median temperature of the area of the orbital cavity in Group 4 (intraocular metastases).

**Table 3:** Mean and standard deviations for temperatures measured at the three areas of affected and normal eyes, grouped according to the types of intraocular tumors

Group		ROI	Variable	Affected			Normal		
				Mean	SD		Mean	SD	
1. Melanoma without treatment (*n*=9)		Central point of the cornea Area of the eye	T Mean	34.25^a^ 34.60	0.78 0.85		33.75^b^ 34.38	0.89 0.76	
			SD	0.84	0.76		0.63	0.21	
			Median	34.72^a^	0.68		34.27^b^	0.83	
			Mode	34.72^a^	0.92		34.00^b^	1.00	
		Area of the orbital cavity	Mean	34.49^a^	0.64		34.13^b^	0.58	
			SD	1.12	0.39		1.01	0.15	
			Median	34.62a x y	0.70		34.04^b^	0.64	
			Mode	35.26^a^	0.65		34.23^b^	1.21	
	2. Melanoma after treatment (relapse) (*n*=4)	Central point of the cornea Area of the eye	T Mean	34.13 34.83	1.10 0.46		33.15 34.25	0.97 0.46	
			SD	0.85	0.36		0.91	0.39	
			Median	34.86	0.68		34.14	0.61	
			Mode	34.87	1.58		34.04	1.41	
		Area of the orbital cavity	Mean	34.96	0.53		34.52	0.24	
			SD	1.04	0.26		1.14	0.17	
			Median	35.09^x^	0.71		34.61	0.42	
			Mode	36.33	0.40		34.98	1.20	
3. Melanoma after treatment (regression) (*n*=4)		Central point of the cornea Area of the eye	T Mean	33.37 34.20	1.38 0.98		34.71 35.10	1.19 1.18	
			SD	0.86^a^	0.34		0.54^b^	0.24	
			Median	34.13	1.05		35.09	1.20	
			Mode	33.40	1.34		35.22	1.32	
		Area of the orbital cavity	Mean	33.70	0.23		34.58	1.38	
			SD	1.36	0.39		0.95	0.44	
			Median	33.82^z^	0.24		34.70	1.36	
			Mode	34.27	1.35		35.27	1.66	
	4. Metastasis (*n*=12)	Central point of the cornea Area of the eye	T Mean	33.23 34.02	1.06 0.89		33.37 34.30	0.99 0.75	
			SD	0.80	0.23		0.83	0.33	
			Median	33.99	0.98		34.28	0.81	
			Mode	33.73	1.12		34.52	0.87	
		Area of the orbital cavity	Mean	34.28^a^	0.82		34.51^b^	0.76	
			SD	1.06	0.21		1.01	0.13	
			Median	34.37a x y z	0.93		34.62^b^	0.82	
			Mode	34.88	1.28		35.33	1.24	
5. Retinal capillary hemangioblastoma (*n*=8)		Central point of the cornea Area of the eye	T Mean	33.40 34.13	1.04 0.81		33.14 33.99	1.44 1.10	
			SD	0.80	0.24		0.80	0.33	
			Median	34.13	0.94		33.92	1.16	
			Mode	34.14	1.08		33.36	1.46	
		Area of the orbital cavity	Mean	34.29	0.68		34.08	0.71	
			SD	1.08	0.35		1.09	0.39	
			Median	34.32^y z^	0.81		34.10	0.78	
			Mode	35.07	1.30		34.94	1.30	

Differences among groups calculated only for the median temperature.

a, b = different superscript letters within rows indicate significant differences (*p*≤0.05),

x, y, z = different superscript letters within columns indicate significant differences (*p*≤0.05). Median denotes the middle value in a series arranged from the lowest to the highest, separating the same number of observations on both sides. Mode denotes the value that appears most often or the value that is most likely to be sampled. T= temperature of a single pixel; SD= standard deviation.

A significant effect of the investigated factors on the median temperature was only noted in subgroups distinguished based on the presence of the intraocular tumor and the type of tumor (p≤0.05). There were significant differences in the median surface temperature of the orbital cavity between Group 1 (untreated uveal melanoma) and Group 3 (uveal melanoma after successful brachytherapy), as well as between Group 2 (uveal melanoma after unsuccessful brachytherapy), Group 3 (uveal melanoma after successful brachytherapy), and Group 5 (retinal capillary hemangioblastoma) (p≤0.05).

## DISCUSSION

According to the analysis of the available literature, the mean temperature of the surface of the eye was 34.02°C ± 0.22 (standard deviation [SD]). There was no significant difference found in temperature between the right and left eyes, or between males and females^(6)^. There was no correlation between the thickness and density of the cornea, the length of the anterior chamber of the eye, and the temperature of the surface of the eye^(7)^. Other studies did not report differences in the thermographic analysis between the left and right sides of the face^(8)^. It is also thought that the physical characteristics of patients (i.e., body hair, obesity, and skin lesions) exert a limited effect on the acquired thermograms due to the analyzed body region^(9)^.

The analysis of demographic data in the studied groups revealed that intraocular hemangiomas were usually detected in younger subjects at a mean age of 44.5 years (SD: ±22.05) and more often in females; metastases and intraocular melanomas were more frequent in elderly subjects. The mean age at diagnosis was 67.92 years (SD: ±12.77) and 73.89 years (SD: ±9.78) for intraocular metastasis and uveal melanoma, respectively. Intraocular metastases and melanoma were more frequent in females and males, respectively (Table 1).

In the examined group, melanoma in the ophthalmoscopic examination appeared as a dark brown tumor, usually located in temporal quadrants; metastases appeared as a bright, off-white tumors. In the ophthalmoscopic examination, retinal hemangioblastoma appeared as a red-orange tumor that lifted the retina, usually with a diameter of 1-1.5 dd (size of the optic disc), occasionally accompanied by retinal exudate, exudative retinal detachment, and dilated and tortuous retinal vessels.

### Uveal melanoma

There are single reports regarding the use of thermography as a diagnostic method for intraocular tumors. In 1971, Kruszewski reported that certain cancers (e.g., uveal melanoma or vascular tumors) are visualized in thermography as hot lesions^(10)^. This hypothesis has been supported by other investigators who observed higher corneal surface temperature in melanoma of the uvea and conjunctiva compared with the normal eyes^(11)^. Santa Cruz et al. found higher temperature by approximately 2-4 K in cutaneous melanoma compared with normal skin^(12)^. Other researchers reported the suitability of thermography for the differentiation between melanoma and benign cutaneous tumors with a size >15 mm^(13)^. Uveal melanoma on CDI examination is characterized by a greater mean maximum blood flow in the central retinal artery and posterior ciliary arteries compared with benign orbital cavernous hemangioma and eyes without any pathology^(14)^.

There are also studies indicating that melanomas are tumors with significant thermal activity, which is caused by neoangiogenesis and abnormal vessel morphology in the tumor mass and the high metabolic activity of tissues^(15)^. Yang et al. reported increased pulsatile ocular blood flow and total choroidal blood flow in eyes with uveal melanoma^(16)^.

There are also reports emphasizing the significant role of the microenvironment and immune system in the progression of melanoma^(17)^. Uveal melanoma is a tumor that secretes macrophage pro-inflammatory cytokines, triggering an inflammatory reaction^(1)^. Studies on melanoma have demonstrated that chemokines, e.g., growth-regulated oncogene α (GROα)/CXCL1, GROβ/ CXCL2, GROγ/CXCL3, and interleukin-8 (IL-8)/CXCL8, control the proliferation of cancer cells^(18)^. Another study revealed increased levels of pro-inflammatory and pro-angiogenic cytokines, such as IL-6, IL-8, interferon gamma-γ, monocyte chemoattractant protein-1, and vascular endothelial growth factor, in eyes with uveal melanoma compared with controls^(19)^. A relationship between cancer and inflammation was first described in 1863 by Virchow. Since then, numerous studies have confirmed the impact of chronic inflammation on the progression and growth of melanoma^(20)^.

Our study supports the above observations. Examination using a thermal imaging camera indicated that eyes in patients from Group 1 were characterized by higher temperature compared with the fellow normal eye of the patient in the range of all measured parameters in regions of interest (i.e., central point of the cornea, area of the eye, and orbital cavity) and lower minimum temperature. Significant differences were found in the mean temperature at the central point of the cornea, median, and mode temperature of the eye area and mean, maximum, median, and mode temperature at the orbital cavity area (Table 3). This is most likely related to the fact that melanoma is characterized by high local vascular density, which ensures the supply of oxygen and nutrients necessary for tumor growth^(21)^.

Interestingly, in the group of patients with melanoma after brachytherapy (Group 2), we found higher values of the mean temperature at the central point of the cornea, mean, maximum, median, and mode temperature in the area of the eye and orbital cavity in four patients treated unsuccessfully. However, lower minimum tempera ture was recorded in eyes with diagnosed melanoma.

In patients from Group 3 with tumor regression, all measured parameters were lower in the affected eye. However, statistical analysis did not reveal any significant differences between these variables, which may be attributed to the small group size (Table 3).

The lower values of the analyzed variables may indicate insufficient blood supply to the retinal areas in the affected eye caused by radiation therapy. This effect leads to retinal occlusion, ischemia, partial retinal atrophy, and the formation of scar tissue^(22)^. Features that may indicate successful therapy include a reduced number of vascularized areas, increased vascular resistance, and increased tumor echogenicity^(23)^. Reduction in peak systolic frequency was reported for choroidal melanoma treated with episcleral brachytherapy. The vessels in the orbital cavity also receive a certain dose of radiation during the treatment of uveal melanoma, which may cause ischemia within the healthy orbital vessels and a lower surface temperature in this region (Table 3). Some researchers presume that the developing radiogenic vasculopathy of the small orbital vessels is the cause of increased vascular resistance^(24)^. This hypothesis was confirmed by other researchers, who revealed a decreased blood flow velocity in the central retinal artery and an increased resistance index in small ocular arteries during the 2-year follow-up of eyes with choroidal melanoma after stereotactic radiotherapy using the Gamma Knife^(25)^.

### Choroidal metastases

Currently, there are no studies on thermal emission in eyes with choroidal metastases. According to Konstantinidis et al., metastatic tumors are rather poorly vascularized^(26)^. The metastatic tumor uses blood vessels at the target tissue, which are necessary for metastatic growth in the distant organ. The anatomy and course of vessels are similar to those of the primary tumor from which the metastasis arises^(21)^.

Our study revealed lower temperatures in the range of all tested parameters and areas in eyes with choroidal metastases. The analysis revealed significant differences in the maximum temperature of the eye area and the mean and median temperatures of the orbital cavity (Table 3). Metastatic tumors most likely develop within the vasculature of the affected site. We suggest that metastatic tumor tissue receives blood and nutrients from the vascular membrane around the lesion, and high metabolic activity is limited to a small area of the metastasis. This may explain the similar temperature measured for the entire ocular area to that of the normal eye. However, the mechanism involved in this process is not entirely clear. Recent reports suggest that if the metastasis is located in well-vascularized areas, the tumor may not create its own system of blood vessels but instead use the existing local vasculature. These reports present different variants of vasculature for metastatic tumors, which may also be other than neo-angiogenic, as in non-small-cell lung carcinoma^(27)^. Ocular metastases may be low-metabolic metastases or secondary avascular tumors; therefore, they do not increase eyeball metabolism, but they could take activity from the choroid around them.

This effect may also be caused by the characteristics of secondary neoplasm growth and compression on adjacent vessels (e.g., retinal arteries) causing ischemia (tissue pushing) or by vasoconstriction of conjunctival blood vessels, which are all consequences of a decrease in ocular surface temperature. Further research is warranted to confirm this trend in a greater number of cases.

### Retinal capillary hemangioblastoma

According to the literature, cutaneous hemangiomas are benign vascular tumors characterized by a greater perfusion compared with normal skin, resulting in increased thermal emission observed using a thermal imaging camera^(28)^. There are reports regarding the use of thermography for monitoring and assessing the treatment of proliferative infantile hemangiomas with beta blockers^(29)^ and predicting their growth. Other studies revealed a median initial temperature of 36.7°C for stable hemangiomas, 37°C for the slightly growing group, and 37.4°C for the growing group^(30)^.

In our study, eyes with diagnosed intraocular hemangioma (Group 5) were characterized by higher parameters for the regions of interest, except for the minimum temperature versus eyes without this pathology; however, there were no significant differences between the analyzed variables.

Despite the small size of the study group, the infrared investigation of intraocular tumors appears to be an interesting concept. Information regarding the tu mor vasculature obtained at an early stage of the diagnostic process can provide valuable diagnostic and prognostic indications. Analysis of larger groups of patients may help assess the efficacy of brachytherapy against melanoma.

The limited availability of highly specialized tests and lack of insight into the fundus of the eye caused by secondary complications of tumor growth have stimulated the search for easily available and rapid methods for the assessment of features of intraocular tumors. Diagnostic tests, such as FA and indocyanine green angiography, are invasive techniques and provide subjective assessment, depending on the experience of the investigator. Moreover, histopathological biopsy is not always feasible. On the other hand, Iveković et al. indicated that CDI, despite its noninvasive nature, may provide biased findings with a significant error. The inaccuracy of CDI may be attributed to the fact that the velocity of blood flow in the cancerous vessel is never measured exactly at the same point, the direction of blood flow is frequently unidentifiable, and the angle between the tested vessel and the probe is often >60°^(14)^.

Thermography is a helpful method, especially when fundus examination of the eye is not possible, due to corneal opacity, a mature cataract, or vitreous hemorrhage. These disorders do not have their own vascularization; hence, they do not cause thermal emission disturbances arising from uveal tumors. The evaluation of thermograms is not dependent on the experience of the examiner, because the camera software independently determines the temperature of the tested area. Potential limitations of ocular thermography include measurement error, lack of standardization during image acquisition (different angle, distance, and environmental conditions), and the failure to exclude other ocular diseases that may affect the test result.

Thermography is an undervalued diagnostic technique; after developing relevant standards for image acquisition, it could be used for the screening and differential diagnosis of intraocular tumors, as well as for the assessment of therapeutic efficacy. Currently, it has been replaced by invasive angiographic or histological examinations. Owing to its simplicity and cost-effectiveness, thermography can be used in specialized ocular oncology centers. The authors intend to conduct further studies on the diagnosis and monitoring of intraocular tumors using thermography and compare this method with other imaging tests. This comparison is expected to determine not only the role of thermography in the diagnosis of intraocular tumor but also the usefulness of thermography in monitoring treatment results. Thermography may become an important complementary or monitoring study of treatment outcomes.

A thermographic examination of the eye and orbital cavity can be used as an additional first-line diagnostic tool for differentiating intraocular tumors.

Thermography can be a helpful tool in monitoring the treatment outcome in patients with intraocular melanoma.

Uveal melanoma prior to treatment is visualized as a hot tumor in thermography, which may indicate its increased vascularization and metabolism.

Intraocular metastases do not appear to be hot tumors in thermography, suggesting their lower vascularization compared with that of uveal melanoma.
